# MetaSim—A Sequencing Simulator for Genomics and Metagenomics

**DOI:** 10.1371/journal.pone.0003373

**Published:** 2008-10-08

**Authors:** Daniel C. Richter, Felix Ott, Alexander F. Auch, Ramona Schmid, Daniel H. Huson

**Affiliations:** 1 ZBIT- Center for Bioinformatics Tübingen, University of Tübingen, Tübingen, Germany; 2 Boehringer Ingelheim Pharma GmbH & Co. KG, Biberach, Germany; NERC Centre for Ecology and Hydrology, United Kingdom

## Abstract

**Background:**

The new research field of metagenomics is providing exciting insights into various, previously unclassified ecological systems. Next-generation sequencing technologies are producing a rapid increase of environmental data in public databases. There is great need for specialized software solutions and statistical methods for dealing with complex metagenome data sets.

**Methodology/Principal Findings:**

To facilitate the development and improvement of metagenomic tools and the planning of metagenomic projects, we introduce a sequencing simulator called MetaSim. Our software can be used to generate collections of synthetic reads that reflect the diverse taxonomical composition of typical metagenome data sets. Based on a database of given genomes, the program allows the user to design a metagenome by specifying the number of genomes present at different levels of the NCBI taxonomy, and then to collect reads from the metagenome using a simulation of a number of different sequencing technologies. A population sampler optionally produces evolved sequences based on source genomes and a given evolutionary tree.

**Conclusions/Significance:**

MetaSim allows the user to simulate individual read datasets that can be used as standardized test scenarios for planning sequencing projects or for benchmarking metagenomic software.

## Introduction

Metagenomics is based on the isolation and characterization of DNA from environmental samples without the need for prior cultivation of microorganisms. In contrast to single genome studies, analyses are applied to entire communities of microbes instead of only few isolated organisms. It has already led to exciting insights into the ecology of different habitats such as ocean [1], soil [2], acid mine [3], human and mouse gut [4], [5] and even into ancient DNA [6].

The research field of Metagenomics is spurred by the recent development and improvement of next-generation sequencing technologies like Roche's 454 pyrosequencing [7]. Although these high through-put technologies promise faster and relatively inexpensive generation of reads, Sanger sequencing still has been used in environmental genome projects [5] to avoid the drawbacks of shorter read lengths.

In general, studies show that algorithms developed for single-genome assembly are only suitable for environmental sequences under special conditions, for example in low complexity populations [2], [8]. In particular, it is very difficult to assemble reads from highly diverse ecologic systems [9]. The problem is that the arrangement of reads into contigs fails or is misleading because contigs are put together from reads from many different genomes.

Currently, the primary goals of metagenomic studies are the investigation of the phylogenetic composition of the sample (taxonomical binning, “Who is out there”), the quantitive analysis (“How many are there?”) and the prediction of genes and their functions (functional binning, “What are they doing”). Since the amount of comparable environmental data is rapidly growing, comparative studies of multiple metagenomic data sets are of great interest as wells. As of September 2008, 44 metagenome studies have already been conducted whereas 86 projects still are on-going [10].

Common strategies for taxonomical binning are for example: (1) detecting phylogenetic markers like *rRNA*, *RecA*, heat shock protein (*HSP70*) and elongation factors (*EF-Tu, EF-G*) [11], (2) comparing reads against a reference database such as NCBI-nr [12] and then analyzing the matches to place the reads in the NCBI taxonomy [13] and (3) measuring the oligonucleotide frequency caused by codon usage or restriction-site frequency [14]–[18].

When it comes to functional binning, sequences are compared to known protein functions, families and pathways provided by several databases, for example COG, KEGG, PFAM, SEED, STRING and TIGRFAM [19]–[24]. A *de novo* search for (unknown) functional units is only feasible if either long reads or contigs are available for the detection of open reading frames.

Another challenge in metagenomic studies is the development of robust statistical techniques [25]. Particularly with regard to comparative metagenomics dealing with highly variable data, these techniques are considered as indispensable for a well-founded analysis.

Despite the enormous amount of sequence data that was generated and analyzed in the past few years, the number of publicly available software specialized in metagenomic data analysis is surprisingly low. Hence, many studies still make use of classic methods, software or web services that originally were not intended for metagenomic data analysis and have to be adapted or pipelined to produce the desired results [8].

Thus, there is a great demand for specialized metagenomic software supporting the analysis process. Because of the complexity of metagenomic data, it is crucial to benchmark new and existing software with standardized test cases using simulated and verifiable data. A first study [9] provides three data sets with varying complexity by selecting original sequence reads from 113 isolated genomes. In their paper, the authors anticipate that these data sets will be used as standard test cases for software testing.

Some other publications already applied the software ReadSim (pre-version of MetaSim, *unpublished*) to generate simulated read data sets for testing their software [18], [26].

### Description of MetaSim

MetaSim takes as input a set of known genome sequences and an abundance profile. This profile determines which genome sequences are selected for the simulation and the relative abundance of each genome sequence in the dataset.

MetaSim integrates an ”induced tree view” of the NCBI taxonomy [27] that can be used to interactively select taxa and inner nodes of the taxonomy to configure their relative abundances. Additionally, the user is able to simulate an ”evolved” population of a single genome sequence, using a population simulator. This feature is aimed at simulating the common real world situation that many different, but closely related strains of a lineage coexist in the same habitat.

Finally, for the construction of a realistic read data set, MetaSim includes a versatile read sequencing simulator. The user is able to choose from different (adaptable) error models of current sequencing technologies (e.g. Sanger [28], [29], Roche's 454 [7] and Illumina (former Solexa) [30]).

MetaSim allows one to construct verifiable read data sets, and additionally, metagenomes variable in size, taxonomical composition and abundance to reflect the diverse and complex output of real metagenomic studies. The resulting data sets can be used to plan and design metagenomic studies and for evaluation and improvement of metagenomics software tools, statistical methods or assembly algorithms.

### Availability

MetaSim is written in Java and can be run with a graphical user interface or in command line mode. Installers for Linux/Unix, MacOS X and Windows are freely available from our website at: http://www-ab.informatik.uni-tuebingen.de/software/metasim.

## Methods

MetaSim's processing pipeline consists of several phases:

1. Selection of source genome sequences from the internal database
2. Configuration of the species abundance profile by setting the relative copy number of the genome sequences
3. Sampling sequencing of fragments according to the species abundance profiles
4. Application of technology-specific error models to the fragments to create sequencing reads

### Configuration of Species Abundance Profiles

At the beginning, whole genome sequences available from public database can be stored locally as source sequences in an integrated database. The user specifies the relative abundance of each genome sequence in a text-based profile file. An interesting feature of MetaSim is the possibility of assigning frequency values not only at the species level but also at higher taxonomical levels. For example, if the genus Escherichia is assigned a certain amount of genome copies, this amount is split and applied uniformly to all descendant species whose sequences are available from the internal database.

**Figure 1:**
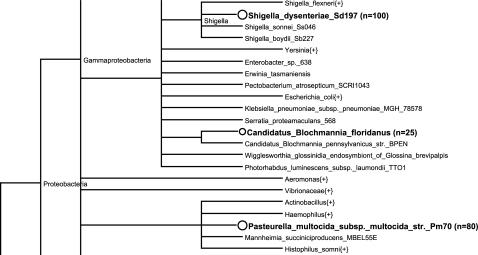
Taxonomy Editor. A clipping of the taxonomy editor view is shown. Three taxa are assigned an abundance value (number in parenthesis). These settings can be either determined in a text-based abundance profile file or directly in the taxonomy editor by right-clicking on a node.

To facilitate this data composition process in GUI mode, MetaSim provides an interactive *taxonomy editor* that visualizes the *induced* NCBI taxonomy, i.e. the genome sequences listed in the profile file are displayed as nodes in a rooted tree (Figure 1). Node sizes reflect the relative number of genome copies for each given taxon.

### Population sampling

The current genome databases reflect only a small part of earth's still unexplored microbial diversity. Thus, a simulated metagenome only based on known genome sequences does not adequately reflect the complexity of realistic data sets.

**Figure 2:**
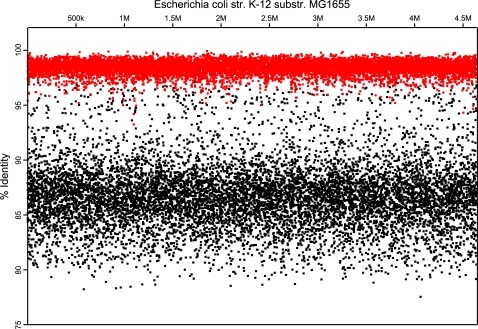
Fragment Recruitment Plot. Black dots represent 10,000 sequencing reads (Sanger technology, ≈800 bp) drawn from 100 evolved offsprings (α = 0.004) of the source genome Escherichia coli K-12 substr. MG1655. Their sequence identity is lower compared to the mapped reads sampled directly form the source genome (red dots).

MetaSim therefore includes a population sampler that optionally generates a set of evolved (mutated) offsprings derived from single source genomes, using a given evolutionary tree. This tree describes how the offspring sequences descend from the source sequence. By default, a random pyholgenetic tree is generated under the Yule-Harding model [31], [32], but alternatively, user-defined trees can also be loaded. As a simple model of DNA evolution, the Jukes-Cantor formula [33] is applied to estimate a probability of change for each base pair, with a customizable transition rate α (0.001 by default) and time *t* based on the edge weights. MetaSim then generates the designated number of evolved genomes and then adds them to the internal genome database. As an example, a fragment recruitment plot (according to [1]) shows 10000 sampled Sanger reads of 100 evolved offspring sequences (α = 0.004) mapped to the source genome (*Escherichia coli K-12 substr. MG1655*) using *blastn* (Figure 2). Read sequences sampled directly from the source genome show a significantly higher identity compared to the mutated sequencing reads.

### Read sampling

MetaSim simulates both Sanger sequencing and Roche's 454 (sequencing-by-synthesis) approach. Additionally, it provides a flexible, empirical error model usable to simulate Illumina's ultra-short reads.

For the simulation of read sequences, statistical approaches are adopted to simulate the distribution of read lengths, its frequency rate and the use of error models depending on the chosen sequencing technology.

**Figure 3:**
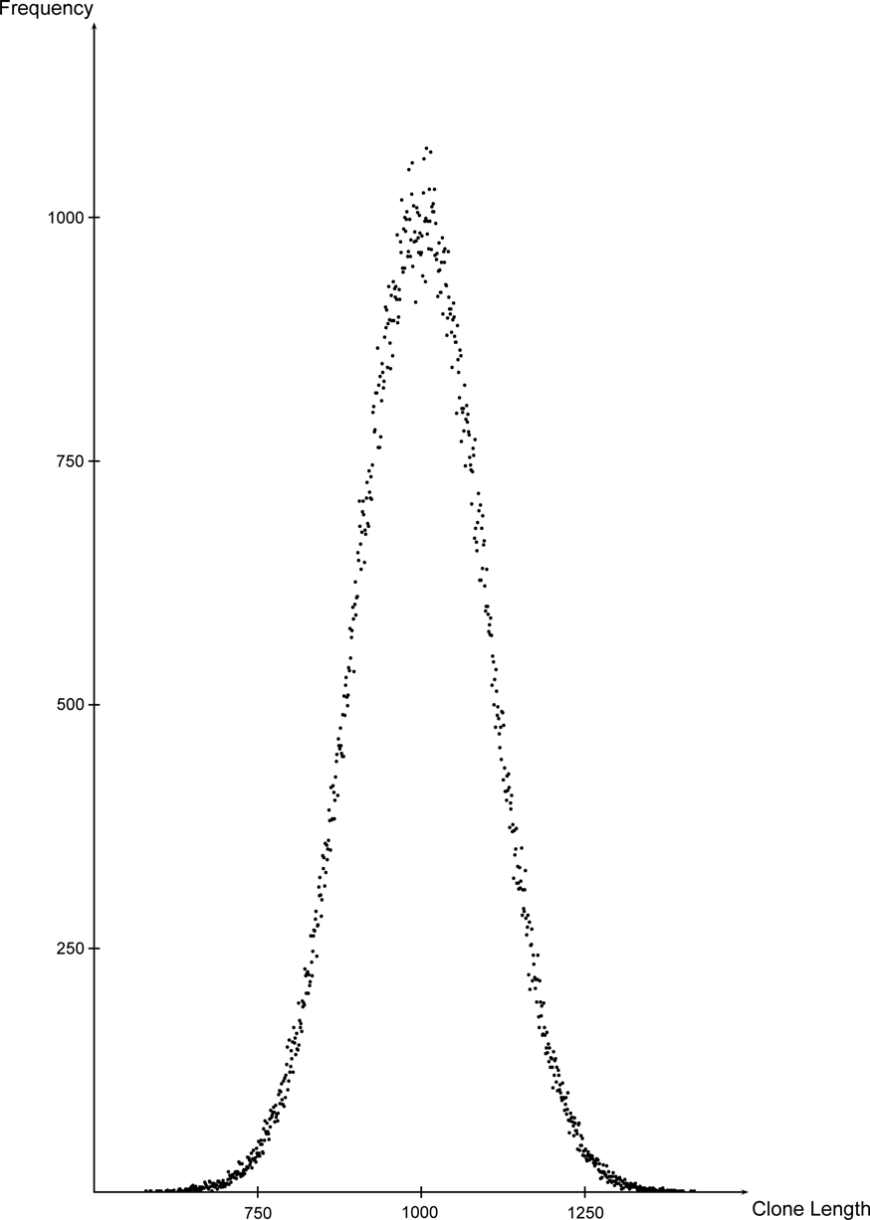
Frequency distribution of clone lengths. As an example, 250,000 clones with mean length 1000 bp and standard deviation of 100 bp were modelled with a normal distribution.

To be able to model mate-pairs as well, MetaSim first extracts large fragments called *clones* from the set of genomes with normally or uniformly distributed lengths. For example, clones with a length of 1000 bp and a standard deviation of 100 bp are modelled with a normal distribution *N*(1000,100) (Figure 3). The overall number of clones is determined by the number of reads or mate-pairs the user desires to generate.

If only one source genome is present in the given profile, the clones are randomly extracted from this single sequence. In contrast, in a typical metagenome simulation, the clones have to be sampled from many genomes of varying length, copy number (e.g. to model the abundance of plasmids versus the organsim genomes) and abundancies.

So, each genome sequence *s* is assigned a weight
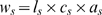
(1)where *l_s_* is the length, *c_s_* is the copy number and *a_s_* is the specified relative abundance of the genome sequence *s* as determined in the profile.

For each length of the clone length distribution, the weights of all sequences are summed up to receive the summarized weight *w_sum_* that is used to compute a sequence probability *p_s_* = *w_s_*/*w_sum_*. Considering the overall lengths distribution, a frequency value for each source sequence is then obtained.

After the clone sampling, the ends of the clones are the basis for the subsequent sampling of the reads or mate-pairs, respectively. Again, read lengths can be either uniformly or normally distributed. Finally, read sequences are processed and modified by applying the selected error model.

### Simulation of Sanger sequencing

A widely-used approach to sequencing large DNA molecules is Sanger sequencing, using a shotgun approach that involves cloning small pieces (or inserts) of DNA and then determining their sequence using fluorescent dideoxynucleotides for termination and capillary electrophoresis.

To simulate Sanger sequencing, we closely followed the implementation of celsim reported in [34]. Each read is subjected to a linearly increasing error rate. We model fixed percentages of deletion errors, insertion errors and substitutions. Further, the simulator is capable of modeling mate-pairs and one can specify the length distribution of inserts.

### Simulation of sequencing-by-synthesis

In pyrosequencing, the intensity of emitted light is used to estimate the length of *homopolymers*, i.e. runs of identical nucleotides in a sequence. During sequencing, the four DNA composing nucleotides are periodically *flowed* over the inserts to be sequenced. Within each flow, the intensity of the signal emitted (which is linear up to 8 bp) reflects the number of nucleotides incorporated. Thus, the addition of a single base or even homopolymer stretches of multiple bases in a single flow can be detected.. For chemical and technical reasons, this signal is subject to fluctuations that lead to sequencing errors. In [7], an error rate of about 3% is reported.

Let *r* denote the length of a given homopolymer. We model the emitted light intensity using a normal distribution *N*(*μ*, *σ*), with mean *μ* = *r* and standard deviation 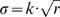, where *k* is a fixed proportionality factor. Following [7], by default we use *k* = 0.15. Although basic statistics implies that the standard deviation should grow with the square root of *r*, in [7] the standard deviation of the light intensity emitted during 454-sequencing is reported to be *σ* = *k*·*r*. Both variants of the calculation are implemented in our software.

A *negative flow* is a flow of nucleotides in which the sequence to synthesize is not elongated. Light intensities of negative flows follow a lognormal distribution, with mean *μ* = 0.23, and standard deviation *σ* = 0.15, see [7]. A random variable *X* is said to be lognormally distributed, if the random variable ln(*X*) is normally distributed.

We simulate base-calling intensities of negative flows and model the misinterpretation of null-mers as homopolymers of length = 1 (insertion). Our algorithm takes the order of the sequencing flows into account. Since the nucleotides are cyclically flowed in the order T,A,C,G, after a given base only two specific negative flows in a specific order are allowed.

During base-calling the algorithm looks at each homopolymer, generates a *N*(*μ*, *σ*) distributed random variable and, according to this variable, decides which length to set for the observed homopolymer.

The signal intensity space is separated into disjoint intervals by probability density functions of all homopolymer signals. The intersections of the density functions 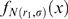, and 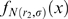 of the normal distributions for different homopolymer lengths *r*_1_ and *r*_2_ are calculated and stored. In each interval one probability density function is maximized. These values are used to decide which homopolymer length is called given a certain signal intensity.

### Simulation of reads with empirical models

As an additional feature, MetaSim includes an empirical error model that allows the incorporation of user-defined error statistics.

The probability of an occurrence of a sequencing error often depends on the position of the erroneous base and its surrounding bases. The program GenFrag [35] originally developed an error model which incorporates different error types (deletion, insertion, substitution) at certain positions with empirical error probabilities.

MetaSim adopts this general approach. The error model is based on mappings (error curves) that assign error rates to specific base positions. Each mapping has three parameters (the last two are optional):

- type of error,
- base at the position where the error occurs and
- base preceding the position where the error occurs.

In this way, 48 independent mappings can be individually determined. In addition, in case of a substitution error the user can specify the probability of integrating a particular base depending on the type of the base at the error position and the preceding base.

Using empirical data, MetaSim provides an error model for the short reads of the Illumina technology.

## Results

**Figure 4:**
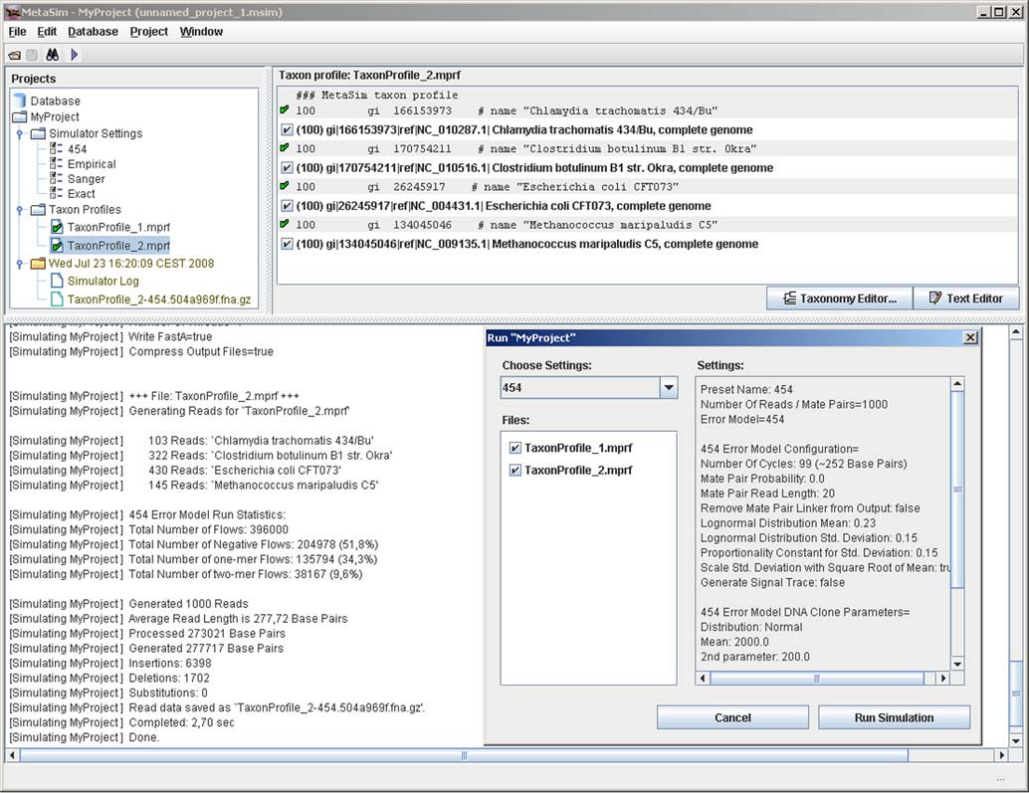
The graphical user interface of MetaSim is divided into three panels: a project tree on the left containing all simulation settings and taxon profiles, an overview and edit panel on the right and a message panel at the bottom. Additionally, a configuration window is shown.

MetaSim can be controlled either by a graphical user interface (Figure 4) or by command line. The command line mode provides access to most of the functions needed for automatic simulation runs. A simulation run generating 400,000 454 reads of length ≈250 bp (a total of 100 Mbp) takes less than 80 seconds on a 2,13 GHz single processor computer.

To show the utility of this simulation software, for example, in the benchmarking of new software, we generated 9 data sets using a range of parameters and used them to test how well the MEGAN software succeeded in successfully binning sequences based on taxonomic classification by homology.

### Simulation study

MetaSim was used to generate three simulated data sets of different species compositions. These data sets were designed and named simLC, simMC and simHC representing low, medium and high complexity communities, respectively (in correspondence to Mavromatis *et al.* [9]). For each data set, three runs were conducted with different sequencing error models and read lengths. Also, each resulting read data set comprises of approximately 15 Mbp, so the number of reads differ accordingly: for the 454 sequencing technology with read length ≈100 bp (≈250 bp), 150,000 (60,000) read sequences were generated. The third data set consists of Sanger reads (≈800 bp) and consisted of 18,750 reads. For a list with all simulation parameters of MetaSim see Supplementary Information Text S1.

Table 1–3 show the relative abundancies and the resulting number of sampled reads for each selected taxon. The simLC data set (Table 1) consists of two taxonomically distant microbes whose relative abundance values differ significantly. The simMC data set (Table 2) is composed of nine microbial species (all from the phylum γ-Proteobacteria) with two dominant populations. The simHC data set (Table 3) consists of 11 diverse microbial species covering several phyla from the superkingdom bacteria. All species in the simHC data set are sampled with the same relative abundance.

**Table 1:** Species abundance and percentage of sampled reads of the simLC dataset.

Abdce	Species	Mbp	454-100^a^	454-250^b^	S-800^c^
**90**	Methanoculleus marisnigri JR1	2.5	82,70	82,61	82,71
**10**	Escherichia coli str. K-12 substr. MG1655	4.6	17,30	17,39	17,29

a 454 technology, 150000 reads (length: 100 bp).

b 454 technology, 60000 reads, (length: 250 bp).

c Sanger technology, 18750 reads, (length: 800 bp).

**Table 2 pone-0003373-t002:** Species abundance and percentage of sampled reads of the simMC dataset.

Abdce	Species	Mbp^a^	454-100^b^	454-250^c^	S-800^d^
**100**	Pseudomonas fluorescens PfO-1	6.4	38,42	38,39	38,13
**100**	Shigella dysenteriae Sd197	4.6	27,07	27,47	27,25
**80**	Pasteurella multocida subsp. multocida str. Pm70	2.3	10,82	10,87	10,81
**50**	Buchnera aphidicola str. APS	6.6	1,97	1,94	1,78
**50**	Francisella tularensis subsp. tularensis Schu 4	1.9	5,69	5,60	5,56
**25**	Alcanivorax borkumensis SK2	3.1	4,68	4,57	4,60
**25**	Candidatus Blochmannia floridanus	7.1	1,03	1,08	1,21
**25**	Pseudomonas entomophila L48	5.9	8,89	8,65	9,26
**5**	Escherichia coli str. K-12 substr. MG1655	4.6	1,43	1,43	1,40

a The length of plasmids is considered as well.

b 454 technology, 150,000 reads (length: 100 bp).

c 454 technology, 60,000 reads, (length: 250 bp).

d Sanger technology, 18,750 reads, (length: 800 bp).

**Table 3:** Species abundance and percentage of sampled reads of the simHC dataset.

Abdce	Species	Mbp^a^	454-100^b^	454-250^c^	S-800^d^
**100**	Agrobacterium tumefaciens str. C58	5.7	11,7	11,7	11,3
**100**	Anabaena variabilis ATCC 29413	7.1	14,7	14,9	14,6
**100**	Archaeoglobus fulgidus DSM 4304	2.2	4,54	4,41	4,55
**100**	Bdellovibrio bacteriovorus HD100	3.8	7,84	7,79	7,83
**100**	Campylobacter jejuni subsp. jejuni 81-176	1.7	3,52	3,6	3,57
**100**	Clostridium acetobutylicum ATCC 824	4.1	8,6	8,56	8,49
**100**	Lactococcus lactis subsp. cremoris SK11	2.6	5,38	5,32	5,54
**100**	Nitrosomonas europaea ATCC 19718	2.8	5,81	5,66	5,59
**100**	Pseudomonas aeruginosa PA7	6.6	13,6	13,6	14
**100**	Streptomyces coelicolor A3(2)	9.1	18,7	18,9	18,9
**100**	Sulfolobus tokodaii str. 7	2.7	5,64	5,59	5,6

a The length of plasmids is considered as well.

b 454 technology, 150,000 reads (length: 100 bp).

c 454 technology, 60,000 reads, (length: 250 bp).

d Sanger technology, 18,750 reads, (length: 800 bp).

Obviously, the amount and ratio of sampled reads in each simulation result reflects the configured abundances and genome sizes. For example, in the simHC data set both *Campylobacter jejuni subsp. jejuni 81-176* and *Streptomyces coelicolor A3(2)* are assigned the same relative abundance of 100. The percentage of sampled reads differs about 15% which can be explained by the difference in genome size (length of contained plasmids are considered as well). Considering all simulations runs, the ratio of sampled reads is almost equal for each species. For example in Table 1, 17,30%, 17,39% and 17,29% reads were sampled for *Escherichia coli str. K12 substr. MG1655* when simulating 454 sequencing technology with read length 100 bp and 250 bp and Sanger sequencing with read length of 800 bp, respectively.

### Taxonomical classification using MEGAN

Following the generation of the nine data sets, a taxonomical assignment of the reads with the MEGAN software was conducted to test its binning functionality. Therefore, all generated read sets were blasted against the NCBI-nr database (downloaded March 2008). MEGAN then assigns these reads to taxa in such a way that the taxonomical level of the assigned taxon reflects the level of conservation of the sampled sequence (Parameter settings of MEGAN: minscore: 0.0, toppercent: 1.0, minsupport: 2, winscore: 0.0).

**Figure 5:**
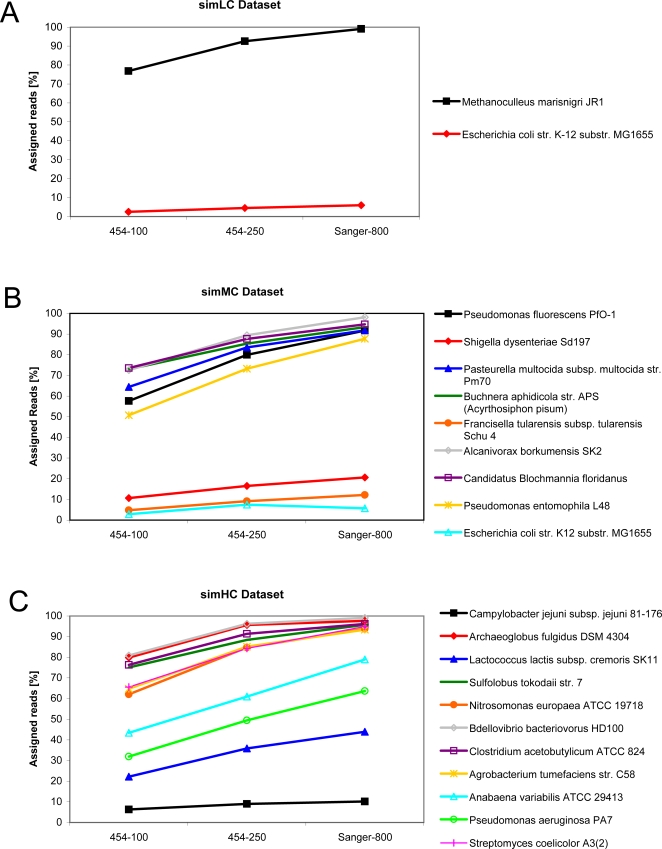
Assignment curves of reads taxonomically classified by MEGAN. The precentage values refer to the number of sampled reads generated for each organism. (A) The simLC dataset consists of only two organisms. The number of assigned reads to M. marisnigri JR1 almost equals the number of its sampled reads whereas E. coli str. K-12 substr. MG1655 has only few assignmentss. (B) In the simMC dataset, the number of assigned reads increases significantly with longer read lengths (except for Shigella dysenteriae Sd197, Francisella tularensis subsp. tularensis Schu 4 and E. coli str. K12 substr. MG1655). (C) In the simHC dataset, the fraction of assigned reads to Campylobacter jejuni subsp. jejuni 81-176, Lactococcus lactis subsp. cremoris SK11 and Pseudomonas aeruginosa PA7 is rather low compared to the other organisms.

The result for each simulation run is shown in Figure 5A–C. (For a list of all results see Supplementary Information, Table S1.) Summarizing all simulations, one observation can be made: The amount of total assignments of reads to taxa correlates with the read length. The longer the read sequence, the more assignments for a taxon are found. This is also true for the correct assignments (true positives) (Supplementary Information Table S1).

Accordingly, the fraction of reads that did not match anything in the NCBI-nr database (% no hits) (Table 4) decreases significantly in case of longer read sequences. These findings were expected since longer read sequences generally give rise to more significant high scoring pairs using BLAST.

**Table 4:** Percentage of assigned, unassigned and “No Hits” reads for all simulation runs.

	Total Reads	%Assigned Reads	%Unassigned Reads	%No Hits^a^
**simLC-454-100**	150000	**83,14**	**0,46**	**16,40**
**simLC-454-250**	60000	**98,58**	**0,85**	**0,57**
**simLC-S-800**	18750	**99,45**	**0,55**	**0,00**
**simMC-454-100**	150000	**81,71**	**0,52**	**17,76**
**simMC-454-250**	60000	**98,08**	**1,02**	**0,91**
**simMC-S-800**	18750	**99,28**	**0,71**	**0,01**
**simHC-454-100**	150000	**81,68**	**0,51**	**17,81**
**simHC-454-250**	60000	**97,55**	**0,93**	**1,52**
**simHC-S-800**	18750	**99,08**	**0,87**	**0,05**

a Reads that did not match anything in the NCBI-nr database.

Independent of the read length, MEGAN is able to assign reads with a high true positive rate. Except for *E. coli str. K12 substr. MG1655* and *Shigella dysenteriae Sd197* in simMC, virtually all reads (98–100%) are correctly classified.

**Figure 6:**
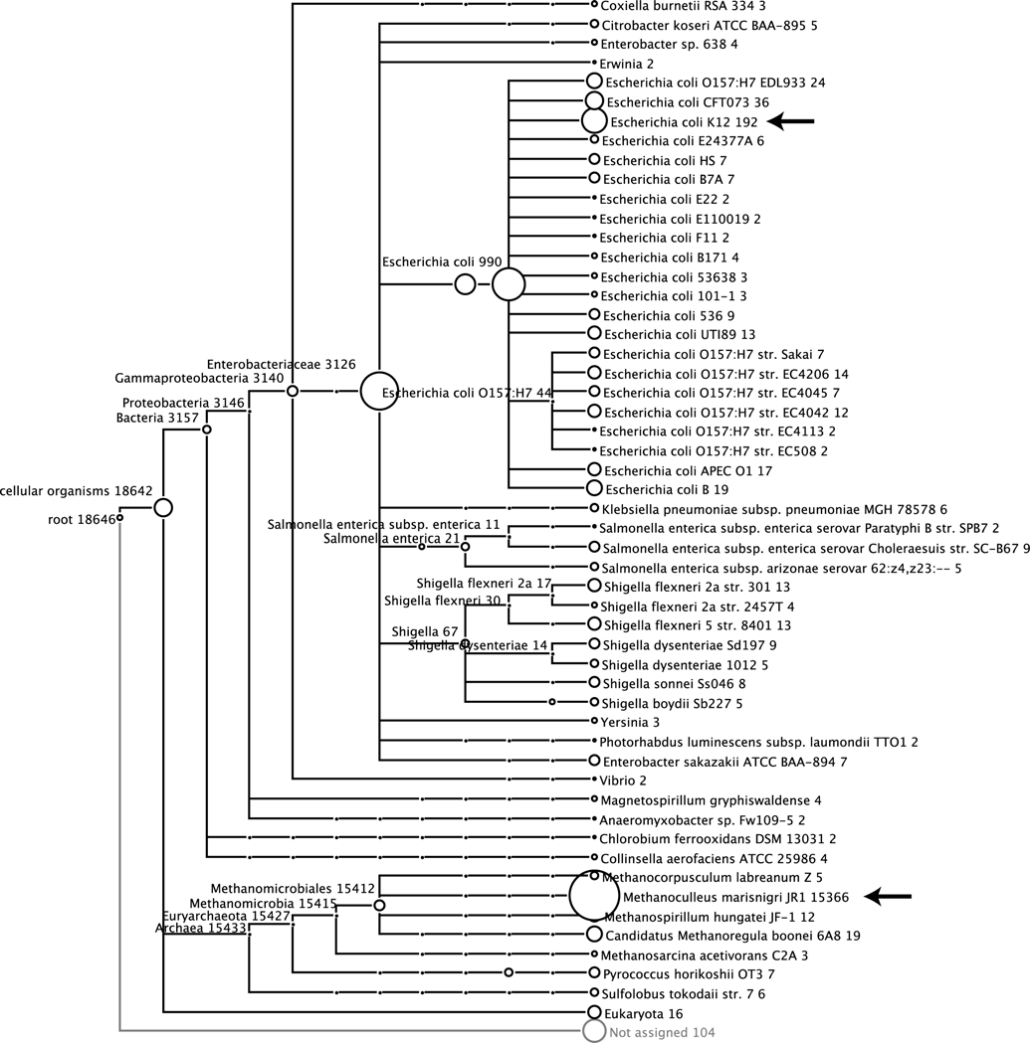
MEGAN visualization of the simLC data set (Sanger technology, read length ≈800 bp). Two arrows point out the two source genomes of the simulation run. The number of assigned reads to E.coli K12 (192) is rather small compared to the number of sampled reads from the genome of E. coli str. K12 substr. MG1655 (192 assigned vs. 3214 sampled reads). Many reads have BLAST hits in multiple strains and clades, so that MEGAN assigns them to an high-order level in the tree e.g. node Bacteria (3157 reads). M. marisnigri JR1 has only few related strains. In this case, the assignment of reads is more specific (15,366 assigned vs. 15,509 sampled reads).

It is important to mention that the composition of the simulated data sets was explicitly intended as an example for a benchmarking study, not for modelling real ecological environments. Thus, simLC consists of only two microbial species that derive from two distinct superkingdoms of the taxonomy (Bacteria and Archae) (Table 1 and Figure 5A). The majority of the sampled reads of *E.coli str. K12 substr. MG1655* were assigned to other taxa and clades in the subtree of Bacteria (Figure 6) leading to false positive hits in MEGAN. However, many closely related strains in the subtree of genus Escherichia were hit supporting the evidence that a high number of genetic functions are shared among them.

A similar problem can be observed in the simMC data set (Figure 5B). To complicate the distinct classification of the reads, all species were taken from the class γ-Proteobacteria. In this example, reads of *S. dysenteriae Sd197*, *Francisella tularensis subsp. tularensis Schu 4* and *E. coli str. K12 substr. MG1655* could not be completely assigned to the correct taxa. However, the high assignment accuracy (correct hits, %TP (Supplementary Information Table S1)) means that practically all assigned reads were correctly classified by MEGAN.

**Figure 7:**
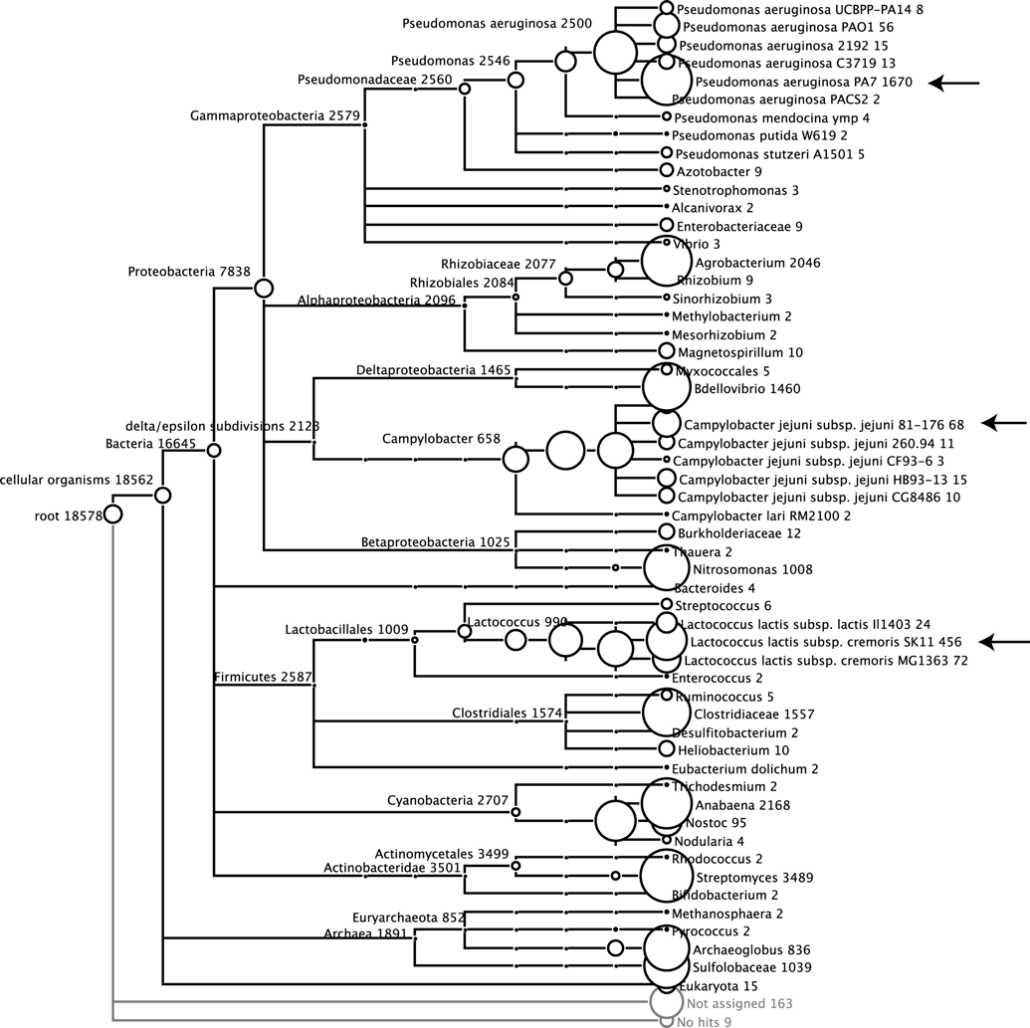
MEGAN visualization of the simHC data set (Sanger technology, read length ≈800 bp). Arrows point out three of the 11 source genomes of the simulation run that show only few assigned reads at species level compared to the number of originally sampled reads. Due to the fact that C. jejuni subsp. jejuni 81-176, L. lactis subsp. cremoris SK11 and P. aeruginosa PA7 share genes with many closely related strains, most of the sampled reads were assigned by MEGAN to an high-order level in the tree (e.g. genus).

Data set simHC contains sequences of many phyla assigned with the same relative abundance of 100 (Table 3). This implies that the amount of sampled reads depends mainly on the length of the source genome sequences. At first sight, the fraction of assigned reads for the three genomes *Campylobacter jejuni subsp. jejuni 81-176*, *Lactococcus lactis subsp. cremoris SK11* and *Pseudomonas aeruginosa PA7* is quite low (10.15%, 43.93% and 63.62% for 800 bp Sanger reads, respectively) (Figure 5C). Though, in the taxonomic tree at genus level (Campylobacter, Lactococcus and Pseudomonas), the number of assigned reads virtually equals the number of sampled reads (Figure 7). This is due to the fact that closely related organisms from the same genus share a lot of genes. Again, the number of correct assignments is almost optimal.

### Summary of MEGAN results

The analysis of the nine artifical data sets help to reveal the pros and cons of taxonomical binning based on homology as done by MEGAN. All simulation runs indicated that MEGAN generally is capable of binning the majority of all generated reads correctly. Additionally, the number of unassigned reads i.e. reads that actually hit sequences in the database but could not be assigned due to MEGAN's parameter settings, is very low. However, reads having many homologous sequences in the database due to high conservation among microbial families and lineages, lead to a rather ”diffuse”, but still correct read assignment: due to gene sharing, MEGAN assigns these reads to high-order taxa closer to the root (e.g. at genus level), thus avoiding probable false-positive assignments (MEGAN uses an LCA-based algorithm [13].). This means that MEGAN classifies those reads rather ”generally” but the assignment still is adequate for interpreting metagenomic data in sufficiency.

Another observation is that the amount of assigned reads correlates strongly with the read length. Obviously, with reads produced by Sanger sequencing (800 bp), taxonomical binning becomes easier compared to short reads (100–250 bp). This oberservation confirms the findings in [13]. Moreover, the positive effect of better assignment and classification of long reads comes with higher costs and workload in the sequencing phase.

The expense for sequencing with Sanger technology is about $500/Mbp (800 bp reads), whereas e.g. pyrosequencing with Roche's 454 technology is only about $100/Mbp (250 bp reads) yielding many more base pairs per run at the same time. On top of the positive cost factor, compared to the Sanger technology, next-generation sequencers do not suffer from cloning bias.

## Discussion

The appearance of next-generation sequencers on the market has boosted the number and scope of (meta-)genomic sequencing projects. A lot of data can be generated in less time demanding fast and precise analysis algorithms and software. However, especially in the field of metagenomics, the problem of producing individual, simulated test cases for benchmarking is open.

We try to fill this gap with MetaSim, a flexible tool for producing simulated read data sets, useful for designing metagenome projects and for testing and comparing metagenomic or assembly software. A lot of parameters can be adapted to generate user defined sequence sets that can serve as verified example data. Currently, the Sanger and Roche's 454 sequencing error model can be selected, as well as the Illumina error model which is based on empirical data. The empirical error model can easily be configured and adapted to other sequencing technologies or error probabilities.

Given this flexibility of the read simulator, it is possible to construct many kinds of individual fragments like, for example reads, contigs or expressed sequence tags (ESTs) derived from existing sequences. We plan to integrate further error models of upcoming sequencers. In addition, future version of MetaSim will extend the population sampler by using more sophisticated models for sequence evolution like HKY [36], as, e.g., already implemented in Seq-Gen [37].

## Supporting Information

Text S1 Parameter settings of MetaSim. For each of the three each simulation runs, the simulation settings are listed.
(0.00 MB TXT)

Table S1 Complete list of simulation results. A complete list of all results of the nine conducted simulation runs evaluated with MEGAN.
(0.04 MB XLS)
